# Challenging selective contracting: reforms for enhancing patient empowerment in healthcare

**DOI:** 10.1186/s13584-025-00673-9

**Published:** 2025-03-06

**Authors:** Gideon Leibner, Devorah Gold, Gabrielle Foreman, Shuli Brammli-Greenberg

**Affiliations:** 1 Faculty of Medicine, Hebrew University-Hadassah, Jerusalem, Israel; 2 Department of Orthopedic Surgery, Hadassah Medical Center, Hebrew University, Jerusalem, Israel

**Keywords:** Selective contracting, Patient choice reforms, Healthcare quality, Socioeconomic, Disparities, Healthcare accessibility

## Abstract

**Background:**

Health insurers and managed care organizations often limit patient choice to in-network care providers through selective contracting, involving procurement agreements with service providers or ownership of healthcare institutions. Patient choice reforms, i.e., reforms which expand hospital choice and reduce the power of the selective contracting, were introduced in a number of countries since the 1990s, in order to address long waiting times and enhance hospital competition based on quality, services, and availability. This study was motivated by Israel’s 2023 health reform, which expanded patient choice by mandating broader hospital choice and enhancing transparency. This study examines reforms in selective contracting models in developed countries and assesses their impact on healthcare quality, accessibility, and socioeconomic disparities.

**Methods:**

A search was conducted on PubMed, Google Scholar, OECD Library, and European Observatory using keywords related to healthcare reform, provider choice, and selective contracting. The search was limited to English-language articles published since 2001.

**Results:**

Traditionally, NHS-based countries did not include patient choice in their systems. Reforms in countries like England and Portugal have since allowed patients choice between hospitals. In contrast, systems with multiple competing insurers, such as Germany, Switzerland, the Netherlands, and Israel, inherently incorporate some patient choice. Israel’s 2023 health reform further broadened hospital choice, while maintaing selective contracting, and enhanced transparency.

Patient choice is influenced by distance, quality, and availability. Patients often prefer nearby hospitals but will travel for higher quality care. Increased hospital competition generally improves care quality but may exacerbate socioeconomic disparities.

Successful components of patient choice reforms include publishing comparative quality indicators and establishing national appointment scheduling systems. These initiatives increase transparency, improve patient decision-making, and drive hospital improvements.

**Conclusions:**

Expanding patient choice in healthcare enhances system efficacy and patient empowerment. However, addressing socioeconomic disparities is essential to ensure equitable access to high-quality care. Future policies should focus on tools and strategies that cater to all patient groups, including accessible and easily understood comprehensive quality assessments and national appointment scheduling systems. Further research should cover a wider range of healthcare systems to understand the challenges and opportunities in patient choice reforms comprehensively.

## Background

Health insurers and managed care organizations (MCO) often limit patient choice of care providers to a network of providers by determining procurement agreements with a limited number of service providers, i.e., Selective Contracting (SC), or by referring to self-owned healthcare institutions such as hospitals, or medical care firms etc. At times, patients may choose a service provider outside the arrangement (outside the network) for an increased deductible price, co-payment or without coverage. Some insurers^1^ provide full coverage solely to in-network care providers and hospitals, whereas others use soft incentives or administrative methods to direct patients to preferred service providers.

Insurers’ preferences for certain providers are sometimes based on the quality of treatment, but more often they stem from procurement agreements that offer significant discounts for services [1, 2].

SC has the potential to enhance efficiency and quality; however, its practical implementation does not always yield these theoretical benefits. Empirical evidence suggests several limitations: (1) Cost Prioritization Over Quality: Insurers often emphasize cost savings rather than service quality. While quality metrics may be part of contract negotiations, enforcement and monitoring mechanisms are frequently inadequate, leading to discrepancies between contractual expectations and actual service delivery [2, 3]. Market Concentration and Monopolistic Behavior: Volume-based contracting can concentrate care in a limited number of hospitals, potentially fostering monopolistic tendencies. This may result in increased patient loads and longer wait times rather than genuine efficiency improvements [3, 4]. Reduced Geographic Accessibility: Insurers may select contracts based on financial incentives rather than ensuring broad geographic coverage. This can disproportionately impact rural and underserved populations, limiting their access to essential healthcare services [5].

In addition, the SC models often lead to competition on the price of services provided on the basis of granting a large treatment volume. Countries with a system based on multiple insurers, such as Germany, Switzerland, the Netherlands, and Israel, make use of SC models to provide large basket of services to citizens, in a reality of limited resources and a need to manage market failures characteristic of healthcare markets [6–8]. National Health System (NHS) based system countries like England, Norway, Portugal, and Denmark make use of some of the components of the SC mechanism [9–11].

However, expanding patient choice has been increasingly viewed as a counterbalance to the limitations imposed by SC mechanisms. While patient decision-making challenges are acknowledged, increasing provider choice does not inherently diminish hospital competition. In healthcare systems with relatively low out-of-pocket costs (such as in Israel), hospitals are incentivized to compete not only on cost but also on factors that influence patient experience, including appointment availability, perceived care quality, and operational efficiency [12, 13]. These factors are often overlooked in selective contracting models that prioritize financial considerations. Since the 1990s, a number of countries, such as England [14], Norway [15], Sweden, the Netherlands, and Denmark [16], have introduced reforms to expand patient choice between hospitals, with the expectation that expanding choice would encourage competition, improve efficiency, and empower patients. In addition, the reforms were consistent with evolving cultural values which emphasized the centrality of choice [17]. While these reforms have demonstrated potential benefits, they also highlight the need to address information asymmetry between patients, hospitals, and insurers. To address the issue of information asymmetry and improve patient decision-making, we advocate for increased transparency through publicly available performance data: Providing access to clinical outcomes, patient satisfaction ratings, and efficiency indicators enables more informed decision-making [18] ;and a national appointment scheduling System: Allowing patients to compare hospital waiting times fosters competition and improves accessibility. These tools are essential to ensure that patients can make informed choices, thereby driving meaningful competition among providers.

Traditionally, in countries with a National Health System (NHS), patient choice was not part of the system. This caused a lack of competition a centralized system, leading to long waiting times, unequal access to care, and disparities in quality of service. As a response, many NHS based systems-initiated reforms to increase patient choice between hospitals. As part of the reforms, England and Portugal invested in developing the supply side by providing incentives to open private hospitals that would compete with public hospitals [9, 19].

In contrast to the NHS-based countries, in multiple competing insurers-based health systems such as Germany, Switzerland, the Netherlands, and Israel, choice is an inherent part of the system and is reflected first and foremost in the choice of an insurer [20]. Insurers often limit patient choice among service providers, including hospitals, [21] by making use of selective contracting. In Switzerland, Germany and the Netherlands, despite the SC practice the networks of providers are expected to be broad due to cultural characteristics that emphasize patient autonomy and right to choose [6, 7, 22].

To summaries, while selective contracting is beneficial for financial sustainability and efficiency, excessive reliance on insurer-led decision-making risks prioritizing cost containment at the expense of quality and accessibility. A more balanced approach, where both insurers and patients exert competitive pressure on hospitals, can lead to more comprehensive improvements in care delivery [23].

### Israel’s selective contracting reform

In 2023, Israel implemented legislative changes to impose revised model that adds restrictions on the Israeli Health Plans (HPs) regarding their process for contracting with hospitals (i.e., selective contracting). These restrictions broadened the choices available to Israeli citizens in need of hospitalization. This reform was driven by increasing public inquiries, high national private health expenditures, a lack of competition among hospitals, and notable disparities in selective contracting across different HPs. [24, 25].

The Israeli state comptroller recommended a change already in 2010, and the German Committee followed him and recommended changes as well in 2012. [24, 26, 27] Under the revised model, the selective contracts between HPs and hospitals are now required to include at least four hospitals, two of which must be located in the patient’s residential area and two of which must be classified as high-capacity tertiary hospitals. [24, 25] Additionally, HPs are mandated to enhance transparency regarding these selection arrangements and are required to list all in-network service providers and their contact information on the payment authorization forms. This ensures that patients are empowered to actively choose their care provider rather than be channeled to service providers with whom the HP has favorable financial contracts. It is important to note that within the framework of this reform, five medical fields (namely: Oncology, IVF services, Mental health hospitalization services, gynecological surgeries, and neurosurgery) are exempt from the SC, allowing patients complete freedom of choice between hospitals.

Following Israel’s SC 2023 reform, this study aims to explore prominent models of selective contracting utilized in developed countries and assess their impact on key characteristics of health systems, such as quality of care, accessibility, and socioeconomic disparities.

The paper provides an overview of major selective contracting models across different health systems, discusses the factors influencing patient’s choice their impact on healthcare quality and access, and evaluates successful international components that could inform Israel’s evolving policy framework**.** Additionally, the study explores the limitations and challenges associated with patient choice reforms, particularly concerning equity and transparency, and concludes with recommendations for improving Israel’s system while ensuring both efficiency and accessibility, in the context of the Israeli reform initiated in September 2023.

## Methods

A methodologic search was conducted on PubMed, Google Scholar, OECD Library, and European Observatory using the following keywords: *healthcare reform*, *provider choice*, *limited contracting*, *selective contracting, patient choice, competition policy, provider competition, outpatient clinics, demand-driven care, managed care, value-driven contracting,* and *hospitalization system*. The search was limited to articles published since 2001 in English.

From the articles that came up in the search, those dealing with selective contracting between medical service providers and insurers/MCOs were selected. In addition, the reference lists of all articles were reviewed. Many articles have been written on the subject, but this study focuses on countries where much research has been conducted, such as Denmark, Norway, Portugal, and England, and countries with a health system similar to Israel, such as Germany, Switzerland, and Slovenia. US-sourced work was excluded because of the fundamentally distinct nature of the healthcare system, which is largely private and operates under different principles compared to the predominantly public or mixed healthcare systems in the focus countries. In total 85 papers were included in the study.

## Results

### Factors influencing patient’s choice

Many factors influence the patient’s choice of service provider in general and hospital in particular, the main ones being: quality, availability, and distance [28, 29]. Hospitals compete on these three parameters to differentiate themselves and attract patients.

#### Distance

Distance is often one of the main characteristics that determines a patient’s hospital default. On some occasions patients are more likely to go to hospitals that are close to their place of residence or located in the area [30–33]. Until the changes in the 2023 regulations mentioned above, Israel was unique in that the nearest hospital was not always in the insured’s HP network. In Switzerland, patients are referred to a hospital in their area of residence (canton) and may receive coverage up to their canton maximum if they apply outside the canton [6] In the other countries we surveyed, there are currently no differences in coverage between areas, but it was found that older patients, non-White people, and those in a lower socioeconomic group tend to choose closer hospitals despite differences in availability and quality [30].

#### Quality

In contrast with the preference for a nearby hospital, there are other occasions when patients are willing to travel to reach higher quality hospitals. It was found in empirical studies from England [34], Germany, and the Netherlands that patients chose to turn to hospitals that have lower complication rates and a better reputation for medically significant procedures such as heart bypass surgeries [28, 35, 36], hip replacements [32], knee replacements [37] and heart catheterization [33]. In the Netherlands, it was found that 38–54% of patients turn to a more distant but higher-quality hospital for elective surgeries in the fields of orthopedics and neurosurgery.

Several studies have explored the impact of hospital competition on the quality of care, with findings indicating that increased competition often leads to enhanced care quality as arises from patient outcomes [30, 36, 38–47]. However, some studies have yielded mixed or inconclusive results regarding the competition-quality relationship [40, 48–52]. Despite research indicating a relationship between competition and improved care quality, the precise mechanisms and potential confounding variables remain unclear, necessitating caution when formulating policies that rely on competition to drive quality improvements. Furthermore, challenges in choice expansion implementation, including development of supply side health provides, loss rates, transparency issues, and disparities in choice utilization, need to be addressed.

#### Availability and accessibility

A review study found that on other occasions patients turned to hospitals with shorter waiting times, [30] which pushed hospitals to shorten the length of queues in order to attract patients. In a complementary study in Israel, it was found that the most significant criterion for a patient choosing a hospital is waiting time [53]. In England, Denmark, and Norway, long waiting times were the impetus for reform legislation to expand choice. In England and Denmark, private hospitals were brought in as service providers for certain procedures and conditions to shorten queues [17]. In countries with a limited private system (e.g. Norway, Netherlands) patients can be referred to service providers outside the country as an alternative to the private system. Similarly, in England and Denmark patients are referred beyond its borders for medical treatment [54].

It was found that following the reforms in the Netherlands and Norway, hospitals have made changes in their practice, such as introducing new services and lengthening the hours of service provision, which led to a decrease in waiting times [55]. However, in a reform to expand choice in Portugal, waiting times increased in quality hospitals. This may have been due to the fact that the reform did not include a mechanism for remuneration according to activity and the payments to hospitals remained as global budgets [56].

### Successful components observed in models implemented in other countries

#### Publication of comparative quality indicators of hospitals

Many countries publish comparative information on the quality of care in hospitals to increase transparency and facilitate patient choice. For example, England established the NHS Choices website, which provides up-to-date and comparative information on the quality of hospitals [57]. As a part of increasing transparency, patients can provide opinions on their experiences in the hospital. Similar quality indicators are available in several countries, such as Switzerland, the Netherlands, Germany, and Denmark, with varying accessibility through government websites or non-profit/commercial channels [15, 17, 22, 31, 58–60].

These reports, although sometimes technically dense, influence patient decision-making and have encouraged improvements in medium-sized for-profit and non-profit supercenter hospitals, [37, 61, 62] following the publication of these indicators. Furthermore, these transparency tools offer benefits, particularly in cost-effective quality improvement scenarios, risk-standardized patient scores, and external oversight, to prevent potential patient selection [63]. These transparency tools strengthen the connection between competition and quality, making them vital for patient-choice expansion reforms.

Moreover, transparency initiatives have the potential to address challenges in healthcare decision-making. For instance, Kuklinski found that the recommendations of patients and the level of expertise of a hospital significantly influence the choice of hospital. This is in addition to metric indicators that also have an impact, although in a less significant way [37]. Similarly, Boonen et al. found that patients often select insurers that don’t limit their service provider options due to a lack of trust in insurers’ ability to choose hospitals effectively [64]. Increasing transparency regarding hospital quality can help overcome this barrier.

Using transparency tools comes with limitations, which in turn pose challenges to maintaining equality of access to care. Firstly, concerns about cream-skimming practices may disproportionately impact vulnerable populations, leaving disadvantaged individuals with limited choices and reduced access to quality care in hospitals with more resources and better reputations. Secondly, patients’ reliance on anecdotal information and word-of-mouth can lead to biased decision-making [65], disadvantaging those with limited access to comprehensive healthcare information, often impacting marginalized or less-informed communities. Thirdly, smaller non-specialized hospitals may struggle to enhance quality due to resource constraints, [61] which disproportionately affect local underserved communities, limiting their access to quality healthcare alternatives and exacerbating healthcare disparities.

#### National appointment systems and wait time transparency

A reform to expand choice allows the patient to contact more hospitals and thus gives him access to more available appointments. To allow patients to examine queues and estimate waiting times in a hospital, health systems in England [66], Portugal [60], and Slovenia [58] have established websites with up-to-date waiting times and/or online national appointment-scheduling systems.

#### Socioeconomic factors of hospital choice

Healthcare choice expansion reforms might increase gaps in healthcare access between different social groups, due to varied usage of the choice options. A study in 2017, which examined 26 papers from the USA, Italy, the Netherlands, England, Sweden, and Canada, showed that people from different social backgrounds chose hospitals differently. While patients are generally more likely to receive treatment from their nearest provider this tendency was more pronounced among older patients, non-White individuals, and those from lower social backgrounds. This trend remained even after considering other factors. As a result, non-White patients often face longer wait times, poorer quality care, and smaller, less technologically advanced hospitals [30]. This pattern, where disadvantaged populations—such as older individuals or those from lower social backgrounds—tend to rely more on the nearest regional hospitals, places these hospitals at a significant disadvantage. As healthier and more mobile patients are more likely to bypass local providers, these regional hospitals may be left to manage a disproportionate share of complex and costly patient needs, and without adequate compensatory mechanisms from the government or insurance companies to address these challenges, these hospitals face financial strain and diminished resources.

[30] Similarly, a study by Keating et al. [67] found that Latino or Black individuals with insurance usually chose nearby hospitals for breast cancer surgery. These hospitals were often lower in quality. The choice was influenced by distance and cultural factors. These hospitals also had more Medicare patients and more Latino or Black patients. Moreover, an English study discovered that after patient choice was expanded, people with higher social status managed to get shorter waiting times by using their social connections or by knowing more about waiting times [68] In addition, regarding the matter of inequality in exercising the right to choose a specialist consultant, Nadine & Clause state that patients with higher levels of education are more likely to exercise this right, and: “*…this relationship is most pronounced in countries that provide a free choice of doctors*” they suggest that investing in *“…careful design of, decision-supporting structures…*” may reduce these gaps [69].

While health care systems publish hospital quality comparisons to help everyone make informed choices, it was found that only specific groups of people use these resources. In Germany, for example, those who looked at hospital quality reports were usually older, better educated, women, and people with long-term illnesses [70]. In the Netherlands, less educated individuals and men were less likely to actively choose their hospital. Some said they depended on their family doctor to make the best hospital choice for them [71].

## Discussion

Autonomy and choice are pivotal in shaping health system policies. The expansion of patient choice in hospitals and throughout the healthcare system, as witnessed over the past thirty years, has been a cornerstone of many international reforms. These reforms aim to foster healthy competition, enhance the quality of medical services, reduce wait times, and hinge on the principle of patient empowerment (German Committee Ministry of Health, 2014).

However, our review reveals a discrepancy in the actualization of this right to choose, which is heavily influenced by socioeconomic factors. This disparity is particularly pronounced among patients from disadvantaged backgrounds, who are less likely to exercise their right to choose. Such trends raise critical questions regarding the efficacy of reforms intended to widen patient choice. These reforms may be inadvertently perpetuating existing healthcare disparities rather than diminishing them.

To effectively bridge this gap, the development and implementation of tools and strategies that cater to significant variables influencing patient choice are essential. These include quality of services, availability of appointments, and proximity to patients’ residences. In terms of service quality, there is a clear need for mechanisms that make comprehensive and understandable quality assessments accessible to all patient groups. This could involve simplifying medical jargon or using various communication channels to obtain broader demographics. Moreover, the establishment of a national booking website or call center for appointment scheduling could be a step towards equalizing access, ensuring that patients from all backgrounds can easily find and book appointments at their hospital of choice.

The issue of geographical distance, often a secondary consideration in policy discussions, also requires attention. Ensuring high-quality care in peripheral and small hospitals is crucial. This would not only address geographical barriers but also support the decentralization of quality healthcare, thereby making it more accessible to patients regardless of their location.

Our study has several limitations, that could impact the interpretation and application of our findings. Our research primarily focuses on countries such as Denmark, England, Germany, Switzerland, and Slovenia, with healthcare systems that are similar in some aspects but quite different in others, from Israel’s health system. This specificity may limit the generalizability of our results to Israeli cases and other healthcare models, particularly those with distinct characteristics, such as the United States. Additionally, our reliance on literature in English published since 2001 might exclude pivotal studies, developments in other languages, or earlier scholarly work. This could potentially overlook significant historical contexts and insights from non-Anglophone research.

The methodological approach, emphasizing selective articles from databases such as PubMed and Google Scholar, could introduce selection bias, favoring certain types of studies or perspectives. This skew may not fully represent the diversity of views and experiences in the field. Furthermore, our study’s reliance on published literature may not capture the real-world complexities faced by patients and healthcare providers. The dynamic nature of healthcare systems and evolving patient preferences suggest that our findings, while informative, might not fully mirror the current or future realities of healthcare choices and their implications.

## Conclusions

In conclusion, while expanding patient choice in healthcare is a crucial step toward empowering individuals and enhancing system efficiency, it is equally essential to address the socio-economic disparities that may emerge from such reforms. Our findings underscore the importance of well-designed policies and interventions that ensure equitable access to quality care for all populations. Key recommendations include developing a transparent and easily navigable information platform, implementing a national online appointment-scheduling system, strengthening peripheral hospitals to decentralize high-quality care, and introducing financial models that support healthcare providers serving disadvantaged communities. Additionally, proactive outreach and education efforts targeting populations with lower healthcare literacy are fundamental to reducing disparities and ensuring that all individuals can make informed healthcare choices.

Moving forward, future research should explore a broader spectrum of healthcare systems, linguistic contexts, and methodological approaches to fully grasp the diverse experiences and challenges in patient choice reforms. A more inclusive and comprehensive analysis will contribute to the development of healthcare systems that are not only competitive and efficient but also fundamentally equitable, accessible, and patient-centered.

## Data Availability

Data sharing is not applicable to this article, as no datasets were generated or analyzed during the current study.

## Abbreviations

MCO: Managed care organizations
SC: Selective contracting
NHS: National Health System

## Footnotes

1 In this paper, we collectively refer to health insurers and Managed Care Organizations (MCOs) as insurers.

## References

[1] Coe E, Leprai C, Oatman J, Ogden J. Hospital networks: Configurations on the exchanges and their impact on premiums. Health International. 2013.

[2] Boone J. Health provider networks with private contracts: Is there under-treatment in narrow networks? J Health Econ. 2019;1(67): 102222. 10.1016/j.jhealeco.2019.102222 31450142

[3] Glied SA, Altman SH. Beyond antitrust: health care and health insurance market trends and the future of competition. Health Aff. 2017;36(9):1572–7. 10.1377/hlthaff.2017.0555 28874483

[4] Dafny L, Ho K, Lee RS. The price effects of cross-market mergers: theory and evidence from the hospital industry. Rand J Econ. 2019;50(2):286–325.

[5] Fortney J, Thill JC, Zhang M, Duan N, Rost K. Provider choice and utility loss due to selective contracting in rural and urban areas. Med Care Res Rev. 2001;58(1):60–75. 11236233 10.1177/107755870105800104

[6] De Pietro C, Camenzind Isabelle Sturny P, Crivelli Suzanne Edwards-Garavoglia Anne Spranger L, Wittenbecher Wilm Quentin F. Switzerland: Health system review. Health Systems in Transition, Vol. 17, 2015. 26766626

[7] Kroneman M, Boerma W, Van Den Berg M, Groenewegen P, De Jong J, Van Ginneken E. Netherlands: Health system review. Health Systems in Transition, Vol. 18, 2016. 27467715

[8] Rosen B, Waitzberg R, Merkur S. Israel: Health system review. Health Systems in Transition, Vol. 17, 2015. 27050102

[9] de Almeida Simões J, Augusto GF, Fronteira I, Hernández-Quevedo C. Portugal: Health system review. Health Systems in Transition, Vol. 19, 2017. 28485714

[10] Okkels Birk H, Karsten V, Andreas R, Allan K, Astrid E, Erica R, et al. Denmark: Health system review. Health Systems in Transition, Vol. 26, 2024.

[11] Tsiachristas A, Vrangbæk K, Gongora-Salazar P, Kristensen SR. Integrated care in a Beveridge system: experiences from England and Denmark. Health Econ Policy Law. 2023;18(4):345–61. 37827835 10.1017/S1744133123000166

[12] Brekke KR, Gravelle H, Siciliani L, Straume OR. Patient choice, mobility and competition among health care providers. In: Health care provision and patient mobility [Internet]. Springer, Milano; 2014 [cited 2025 Jan 13]. p. 1–26. Available from: https://link.springer.com/chapter/10.1007/978-88-470-5480-6_1 10.1007/978-88-470-5480-6_1 24864380

[13] Longo F, Siciliani L, Moscelli G, Gravelle H. Does Hospital Competition Improve Efficiency? The effect of the patient choice reform in England [Internet]. 2017 [cited 2025 Jan 13]. Available from: www.york.ac.uk/che 10.1002/hec.3868 30815943

[14] Cooper Z, Gibbons S, Jones S, Mcguire A. Does hospital competition save lives? Evidence from the English NHS Patient Choice Reforms. The Economic Journal [Internet]. 2011 Aug 1 [cited 2025 Jan 11]; 121(554): F228–60. Available from: 10.1111/j.1468-0297.2011.02449.x 10.1111/j.1468-0297.2011.02449.x PMC4373154 25821239

[15] Brekke KR, Canta C, Siciliani L, Straume OR. Hospital competition in a national health service: evidence from a patient choice reform. J Health Econ. 2021;1(79): 102509. 10.1016/j.jhealeco.2021.102509 34352647

[16] Vrangbaek K, Robertson R, Winblad U, Van De Bovenkamp H, Dixon A. Choice policies in Northern European health systems. Health Econ Policy Law [Internet]. 2012 [cited 2024 Jan 26]; 7:47–71. Available from: 10.1017/S1744133111000302 10.1017/S1744133111000302 22221928

[17] Vrangbaek K, Robertson R, Winblad U, Van De Bovenkamp H, Dixon A. Choice policies in Northern European health systems. Health Econ Policy Law. 2012;7(1):47–71. 22221928 10.1017/S1744133111000302

[18] Hadian SA, Rezayatmand R, Shaarbafchizadeh N, Ketabi S, Pourghaderi AR. Hospital performance evaluation indicators: a scoping review. BMC Health Serv Res [Internet]. 2024 Dec 1 [cited 2025 Jan 13]; 24(1):1–17. Available from: https://bmchealthservres.biomedcentral.com/articles/10.1186/s12913-024-10940-1 10.1186/s12913-024-10940-1 PMC11064245 38693562

[19] Vrangbaek K, Robertson R, Winblad U, Van De Bovenkamp H, Dixon A. Choice policies in Northern European health systems. Health Econ Policy Law. 2012;7:47–71. 22221928 10.1017/S1744133111000302

[20] Van De Ven WPMM, Beck K, Buchner F, Chernichovsky D, Gardiol L, Holly A, et al. Risk adjustment and risk selection on the sickness fund insurance market in five European countries. Health Policy. 2003;65(1):75–98. 12818747 10.1016/s0168-8510(02)00118-5

[21] Propper C. Competition in health care: lessons from the English experience. Health Econ Policy Law. 2018;13(3–4):492–508. 29417915 10.1017/S1744133117000494

[22] Blümel M, Spranger A, Achstetter K, Maresso A, Busse R. Germany: health system review 2020. 2020. 34232120

[23] Enthoven AC. The history and principles of managed competition. Health Aff. 1993;12(suppl 1):24–48. 10.1377/hlthaff.12.suppl_1.24 8477935

[24] Choice arrangements between health funds and hospitals. 2023.

[25] National Health Insurance Regulations—Selection policy among Service Providers (Hebrew). Israel - Ministry of health. 2005.

[26] Financial arrangements in health insurance funds for the general hospitals and the effect on the insured. 2010.

[27] The Advisory Committee for Strengthening the Public Health System. 2014.

[28] Gaynor M, Propper C, Seiler S. Free to choose? Reform, choice, and consideration sets in the English National Health Service. Am Econ Rev. 2016;106(11):3521–57. 29553210 10.1257/aer.20121532

[29] Victoor A, Delnoij DM, Friele RD, Rademakers JJ. Determinants of patient choice of healthcare providers: a scoping review. BMC Health Serv Res. 2012;12(1):1–16. 22913549 10.1186/1472-6963-12-272 PMC3502383

[30] Aggarwal A, Lewis D, Mason M, Sullivan R, Van Der Meulen J. Patient mobility for elective secondary health care services in response to patient choice policies: a systematic review. Med Care Res Rev. 2017;74(4):379–403. 27357394 10.1177/1077558716654631 PMC5502904

[31] Beukers PDC, Kemp RGM, Varkevisser M. Patient hospital choice for hip replacement: empirical evidence from the Netherlands. Eur J Health Econ. 2014;15(9):927–36. 24158316 10.1007/s10198-013-0535-7

[32] Beckert W, Christensen M, Collyer K, Capps C, Cooper Z, Ennis S, et al. Choice of NHS-funded hospital services in England*. Econ J. 2012;122(560):400–17.

[33] Varkevisser M, van der Geest SA, Schut FT. Do patients choose hospitals with high quality ratings? Empirical evidence from the market for angioplasty in the Netherlands. J Health Econ. 2012;31(2):371–8. 22425770 10.1016/j.jhealeco.2012.02.001

[34] Moscelli G, Siciliani L, Gutacker N, Gravelle H. Location, quality and choice of hospital: evidence from England 2002–2013. Reg Sci Urban Econ. 2016;1(60):112–24. 10.1016/j.regsciurbeco.2016.07.001 PMC5063539 27766000

[35] Pilny A, Mennicken R. Does Hospital Reputation Infl uence the Choice of Hospital? #516. Ruhr economic papers. 2014.

[36] Siciliani L, Chalkley M, Gravelle H. Does provider competition improve health care quality and efficiency? Expectations and evidence from Europe policy brief 48 health systems and policy analysis. 2022. 36800879

[37] Kuklinski D, Vogel J, Geissler A. The impact of quality on hospital choice. Which information affects patients’ behavior for colorectal resection or knee replacement? Health Care Manag Sci. 2021;24(1):185–202. 33502719 10.1007/s10729-020-09540-2 PMC8184721

[38] Cooper Z, Gibbons S, Jones S, Mcguire A. does hospital competition save lives? Evidence from the english NHS patient choice reforms. Econ J. 2011;121(554):F228–60. 10.1111/j.1468-0297.2011.02449.x PMC4373154 25821239

[39] Gaynor M, Moreno-Serra R, Propper C. Death by market power: reform competition, and patient outcomes in the National Health Service. Am Econ J Econ Policy. 2013;5(4):134–66.

[40] Propper C, Burgess S, Gossage D. Competition and quality: evidence from the NHS internal market 1991–9. Sour Econ J. 2008;118(525):138–70.

[41] Croes RR, Krabbe-Alkemade YJFM, Mikkers MC. Competition and quality indicators in the health care sector: empirical evidence from the Dutch hospital sector. Eur J Health Econ. 2018;19(1):5. 28050682 10.1007/s10198-016-0862-6 PMC5773634

[42] Ghandour Z, Siciliani L, Straume OR. Investment and quality competition in healthcare markets. J Health Econ. 2022;1(82): 102588. 10.1016/j.jhealeco.2022.102588 35065851

[43] Han L, Boyle JM, Walker K, Kuryba A, Braun MS, Fearnhead N, et al. Impact of patient choice and hospital competition on patient outcomes after rectal cancer surgery: a national population-based study. Cancer. 2023;129(1):130. 36259432 10.1002/cncr.34504 PMC10092598

[44] Shen YC. The effect of financial pressure on the quality of care in hospitals. J Health Econ. 2003;22(2):243–69. 12606145 10.1016/S0167-6296(02)00124-8

[45] Bloom N, Propper C, Seiler S, Van J. The impact of competition on management quality: evidence from public hospitals. Rev Econ. 2015;82(2):457–89.

[46] Moscelli G, Gravelle H, Siciliani L, Santos R. Heterogeneous effects of patient choice and hospital competition on mortality. Soc Sci Med. 2018;1(216):50–8. 10.1016/j.socscimed.2018.09.009 30265998

[47] Gravelle H, Santos R, Siciliani L. Does a hospital’s quality depend on the quality of other hospitals? A spatial econometrics approach. Reg Sci Urban Econ. 2014;1(49):203. 10.1016/j.regsciurbeco.2014.09.005 PMC4375725 25843994

[48] Jiang Q, Tian F, Liu Z, Pan J. Hospital competition and unplanned readmission: evidence from a systematic review. Risk Manag Healthcare Policy. 2021;14:473–89. 10.2147/RMHP.S290643 PMC7873024 33574721

[49] Mukamel DB, Zwanziger J, Tomaszewski K. HMO penetration, competition, and risk-adjusted hospital mortality. Health Serv Res. 2001;36(6 Pt 1):1019. 11775665 PMC1089276

[50] Propper C, Burgess S, Green K. Does competition between hospitals improve the quality of care?: hospital death rates and the NHS internal market. J Public Econ. 2004;88(7–8):1247–72.

[51] Moscelli G, Gravelle H, Siciliani L. Hospital competition and quality for non-emergency patients in the English NHS. RAND J Econ. 2021;52(2):382–414.

[52] Gowrisankaran G, Town RJ. Competition, payers, and hospital quality. Health Serv Res. 2003;38(6 Pt 1):1403. 14727780 10.1111/j.1475-6773.2003.00185.x PMC1360956

[53] Leibner G, Bramli-Greenberg S. Patients choice in the hospital system, knowledge and public preferences [Internet]. 2023. Available from: https://www.israelhpr.org.il/research-abstracts/הסדרי-הבחירה-במערכת-האשפוזית-ידע-והעד/

[54] Glinos IA, Baeten R, Maarse H. Purchasing health services abroad: Practices of cross-border contracting and patient mobility in six European countries. Health Policy. 2010;95(2–3):103–12. 20031249 10.1016/j.healthpol.2009.11.016

[55] Maarse H, Jeurissen P, Ruwaard D. Results of the market-oriented reform in the Netherlands: a review. Health Econ Policy Law. 2016;11:161–78. 26278627 10.1017/S1744133115000353

[56] Barros PP. Quality decreases from introducing patient choice in a National Health Service. Portuguese Econ J. 2022;21(3):351–81.

[57] NHS services - NHS [Internet]. [cited 2024 Jun 20]. Available from: https://www.nhs.uk/nhs-services/

[58] Albreht T, Polin K, Pribaković R, Marjeta B, Mircha K, Petra P, et al. Health system review Slovenia. European Observatory on Health Systems and Policies. Vol. 23, 2021.

[59] Marshall MN, Shekelle PG, Davies HTO, Smith PC. Public reporting on quality in the US and the UK. Health Aff. 2017;22(3):134–48. 10.1377/hlthaff.22.3.134. 10.1377/hlthaff.22.3.134 12757278

[60] Simões J, Augusto GF, Fronteira I. Introduction of freedom of choice for hospital outpatient care in Portugal: implications and results of the 2016 reform. Health Policy. 2017;121(12):1203–7. 28987456 10.1016/j.healthpol.2017.09.010

[61] Strumann C, Geissler A, Busse R, Pross C. Can competition improve hospital quality of care? A difference-in-differences approach to evaluate the effect of increasing quality transparency on hospital quality. Eur J Health Econ. 2022;23(7):1229. 34997865 10.1007/s10198-021-01423-9 PMC9395484

[62] Emmert M, Hessemer S, Meszmer N, Sander U. Do German hospital report cards have the potential to improve the quality of care? Health Policy. 2014;118(3):386–95. 25074783 10.1016/j.healthpol.2014.07.006

[63] Chen Y, Sivey P. Hospital report cards: quality competition and patient selection. J Health Econ. 2021;1(78): 102484. 10.1016/j.jhealeco.2021.102484 34218041

[64] Boonen LHHM, Schut FT. Preferred providers and the credible commitment problem in health insurance: first experiences with the implementation of managed competition in the Dutch health care system. Health Econ Policy Law. 2011;6(2):219–35. 21122187 10.1017/S1744133110000320

[65] Huppertz JW, Carlson JP. Consumers’ use of HCAHPS ratings and Word-of-Mouth in hospital choice. Health Serv Res. 2010;45(6 Pt 1):1602. 20698896 10.1111/j.1475-6773.2010.01153.x PMC3026949

[66] Greenhalgh T, Stones R, Swinglehurst D. Choose and Book: a sociological analysis of ‘resistance’ to an expert system. Soc Sci Med. 2014;1(104):210–9. 10.1016/j.socscimed.2013.12.014 24581080

[67] Keating NL, Kouri EM, He Y, Freedman RA, Volya R, Zaslavsky AM. Location Isn’t everything: proximity, hospital characteristics, choice of hospital, and disparities for breast cancer surgery patients. Health Serv Res. 2016;51:1561–83. 26800094 10.1111/1475-6773.12443 PMC4946041

[68] Moscelli G, Siciliani L, Gutacker N, Cookson R. Socioeconomic inequality of access to healthcare: Does choice explain the gradient? J Health Econ. 2018;1(57):290–314. 10.1016/j.jhealeco.2017.06.005 28935158

[69] Reibling N, Wendt C. Gatekeeping and provider choice in OECD healthcare systems. Curr Sociol. 2012;60(4):489–505. 10.1177/0011392112438333.

[70] Emmert M, Kast K, Sander U. Characteristics and decision making of hospital report card consumers: lessons from an onsite-based cross-sectional study. Health Policy. 2019;123(11):1061–7. 31383371 10.1016/j.healthpol.2019.07.013

[71] Rademakers J, Nijman J, Brabers AEM, de Jong JD, Hendriks M. The relative effect of health literacy and patient activation on provider choice in the Netherlands. Health Policy. 2014;114(2–3):200–6. 23972373 10.1016/j.healthpol.2013.07.020

